# The complete mitochondrial genome of Neon rainbowfish (*Melanotaenia praecox* Weber & de Beaufort, 1922)

**DOI:** 10.1080/23802359.2016.1168715

**Published:** 2016-06-20

**Authors:** Yuming Zhao, Zaizhong Chen, Jianzhong Gao, Lei Wang, Zhe Xu

**Affiliations:** a Key Laboratory of Freshwater Fishery Germplasm Resources, Ministry of Agriculture, Shanghai, PR China;; b Shanghai Collaborative Innovation Center for Aquatic Animal Genetics and Breeding (ZF1206), Shanghai Ocean University, Shanghai, PR China

**Keywords:** *Melanotaenia praecox*, mitogenome, rainbowfish

## Abstract

In this study, the mitochondrial genome of *Melanotaenia praecox* Weber and de Beaufort, 1922(Atheriniformes: Melanotaeniidae) was sequenced for the first time. The assembled mitogenome consisting of 16,536 bp, includes 13 protein-coding genes, 22 transfer RNAs, 2 ribosomal RNAs genes and 1 putative control region. The overall base composition of *M. praecox* is 27.51% for A, 29.97% for C, 16.18% for G, 26.34% for T and shows 89% identities to Lake Kutubu Rainbowfish, *Melanotaenia lacustris*. These data would provide useful molecular information for phylogenetic relationships within the family Melanotaeniidae species.

The Neon rainbowfish (*Melanotaenia praecox*) are bright neon blue with red dorsal, anal and caudal fins. They may reach a maximum size of 8 cm, but are usually less than 6 cm. Males have red-edged fins while the fins of females are pure yellow. *Melanotaenia praecox* is very similar in appearance to *M. rubrivittata*. The two species share a number of similarities. But *M. praecox* lacks the characteristic red body stripes of male *M. rubrivittata*. The males of *M. praecox* also tend to be deeper bodied than males of *M. rubrivittata* (Allen 1991).

Samples of *M. praecox* were wild captured from a tributary of the Mamberamo River in West Papua, then the muscle was dissected and preserved in pure alcohol. The specimens were stored in Fish Specimens Museum in Shanghai Ocean University, the accession number is SHOU20150675001. Then their genomic DNA was extracted from muscle by using Sangon Mag-MK Animal Genomic DNA extraction kit (Sangon, Shanghai, China). The primers were designed according to the complete mitochondrial genome of *Melanotaenia boesemani* (KT380951) deposited in GenBank. PCR amplification and sequencing of the products were performed according to the method described by He et al. (2011) with slight modifications.

The complete mitochondrial genome of *M. praecox* was 16,536 bp in size (GenBank accession no. KU743245), including 13 protein-coding genes, 22 transfer RNAs, 2 ribosomal RNAs genes and 1 putative control region. All protein-coding genes are encoded on H-strand with exception of protein-coding genes of *ND6*. All tRNA genes are encoded on H-strand with exception of *tRNA-Gln*, *tRNA-Ala*, *tRNA-Asn*, *tRNA-Cys*, *tRNA-Tyr*, *tRNA-Ser* (UGA), *tRNA-Glu* and *tRNA-Pro*. All the 13 mitochondrial protein-coding genes share the start codon ATG, except for *COI* (GTG start codon). The stop codon, TAA, is present in *COI*, *ND4L* and *ND5*; TAG is present in *ND1*, *ATP8*, *ND6* and *Cytb*; an incomplete stop codon ‘‘TA–’’ is found in *ND2*, *ATP6* and *COIII*; and ‘‘T– –’’ is found in *COII*, *ND3* and *ND4*. The longest one is *ND5* gene (1839 bp) in all protein-coding genes, whereas the shortest is *ATP8* gene (168 bp). The size of the 22 tRNA ranges from 66 bp to 74 bp. The two ribosomal RNA genes, 12S rRNA gene (946 bp) and 16S rRNA gene (1679 bp), are located between *tRNA-Phe* and *tRNA-Leu* (UAA) and separated by *tRNA-Val*.

The control region, which is 880 bp, is located between *tRNA-Pro* and *tRNA-Phe*. The overall base composition of *M. praecox* is 27.51% for A, 29.97% for C, 16.18% for G, 26.34% for T, which is characteristic of mitochondrial genomes of other bony fish (Miya et al. 2003; Zhao et al. 2015a,b), and shows 89% identities to Lake Kutubu Rainbowfish, *M. lacustris* (Figure 1). We expect that the present result would elucidate the further phylogenetic approach among different species of rainbowfishes.

**Figure 1.**
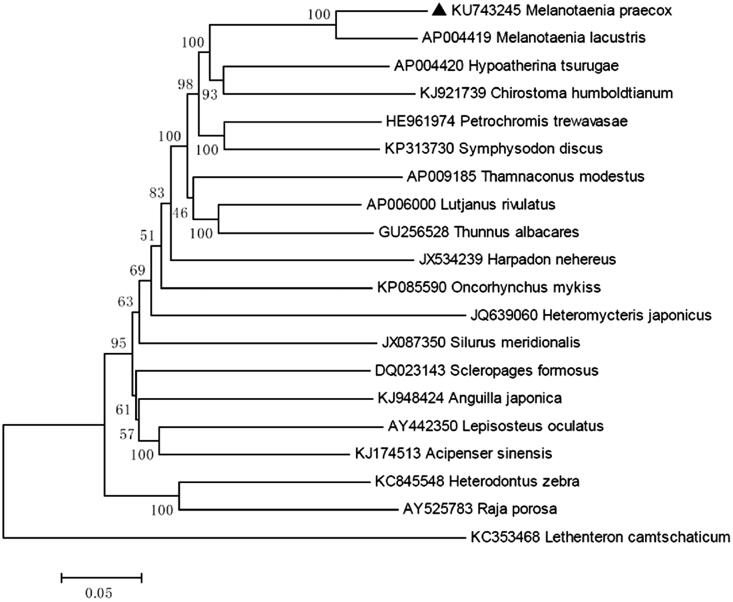
Neighbor-joining (NJ) tree of 20 species complete mitochondrial genome sequence. The phylogenetic relationships of *Melanotaenia praecox* are close with *Melanotaenia lacustris* using *Lethenteron camtschaticum* as an outgroup.
